# A Rare Presentation of Disseminated Peritoneal Leiomyomatosis Following Laparoscopic Hysterectomy and Its Multidisciplinary Management

**DOI:** 10.7759/cureus.75829

**Published:** 2024-12-16

**Authors:** Adhithya N Balaji, Balaji Balasubramanian, Simran Arora, Panna Shetty

**Affiliations:** 1 Internal Medicine, Università Cattolica Del Sacro Cuore, Rome, ITA; 2 Surgery and Oncology, New Medical Centre Specialty Hospital, Abu Dhabi, ARE; 3 Surgery, University of Debrecen, Debrecen, HUN; 4 Pathology, New Medical Centre Royal Hospital, Khalifa City, Abu Dhabi, ARE

**Keywords:** disseminated peritoneal leiomyomatosis, gastrointestinal stromal tumor (gist), gonadotropin-releasing hormone (gnrh) analogue, laparoscopic morcellation, uterine leiomyomata

## Abstract

Disseminated peritoneal leiomyomatosis (DPL) is a rare entity. It is a benign disease but can mimic disseminated malignancy with extensive disease at multiple sites within the abdominopelvic cavity. The primary contributing factor is postulated to be peritoneal spillage of benign leiomyoma, especially after laparoscopic intervention, although hormonal influences might also play a role. It primarily affects females of childbearing age. Cross-sectional imaging studies offer guidance regarding the extensive nature of the disease and surgery is the primary treatment modality. Complete resection, personalized according to the extent of the disease, has to be planned through a multidisciplinary approach. The diagnosis can only be confirmed by histopathology. Hormone suppression might have a role in selected cases.

## Introduction

In this case report, we have discussed a rare case of disseminated peritoneal leiomyomatosis following laparoscopic hysterectomy for uterine leiomyomata (UL). UL is one of the most common causes for surgical intervention in young females in the fertile age group. The smooth muscle tumor has a wide pathological range from benign leiomyoma to smooth muscle tumor of uncertain malignant potential to leiomyosarcoma at the end of the spectrum. Few of them present at atypical locations and with unusual growth patterns. These include intravenous leiomyomatosis, benign metastasizing leiomyoma, and disseminated peritoneal leiomyomatosis (DPL) [1]. Various aspects of the diagnostic and therapeutic challenges during the course of treatment of DPL have been discussed along with literature review. In this era of laparoscopic surgeries, this article is aimed at increasing the understanding of the disease process so that the plan of treatment in such rare cases can be optimized.

## Case presentation

History

A 44-year-old female patient presented to the hospital with abdominal distention in February 2021. She had noticed a gradual increase in abdominal distention for the past six to 10 months and had experienced vague dragging pain for the past few days. There was no vomiting, change in bowel habits, or loss of weight or appetite. She had no known comorbidity. She was married and had two children. She was not on any medication, including hormonal medication. She had no family history of malignancy and had a history of laparoscopic hysterectomy done three years ago for menorrhagia and fibroid uterus. However, her ovaries were not removed. Histopathology of the specimen showed benign changes suggestive of leiomyoma. She underwent an ultrasound (US) scan at another facility, which revealed multiple solid masses in the abdomen and pelvis. Consequently, she was referred for further management.

Clinical examination

The patient's general condition was good with an Eastern Cooperative Oncology Group (ECOG) performance status of 1. Clinical examination of the abdomen revealed large palpable nodular masses in all the quadrants of the abdomen. Laparoscopic scars were noted and nodules were felt at the port sites. There was no tenderness. Pelvic examination revealed normal vaginal and rectal mucosa. A similar nodular mass was noted on rectal examination. Supraclavicular or inguinal nodes were not noted. Breast examination was normal. Mild pedal edema was noticed in both legs. 

Surgical history

The discharge notes of the surgery done in 2018 were reviewed. Her preprocedural US scan revealed multiple fibroids in the uterus, with the largest measuring about 5.6 cm. A laparoscopic hysterectomy procedure was described. During the surgery, the urinary bladder had been opened accidentally and was repaired by the surgeon. There was no mention of spillage of fibroid or the specimen. The histopathology report showed a morcellated specimen of the uterus weighing 680 g and it was reported as benign leiomyoma. 

Other investigations

Basic blood tests including blood counts, renal and liver function tests, and inflammatory markers like CRP were normal. Tumor markers, including cancer antigen 125 (CA 125) and carcinoembryonic antigen (CEA), were within the normal range. 

**Figure 1:**
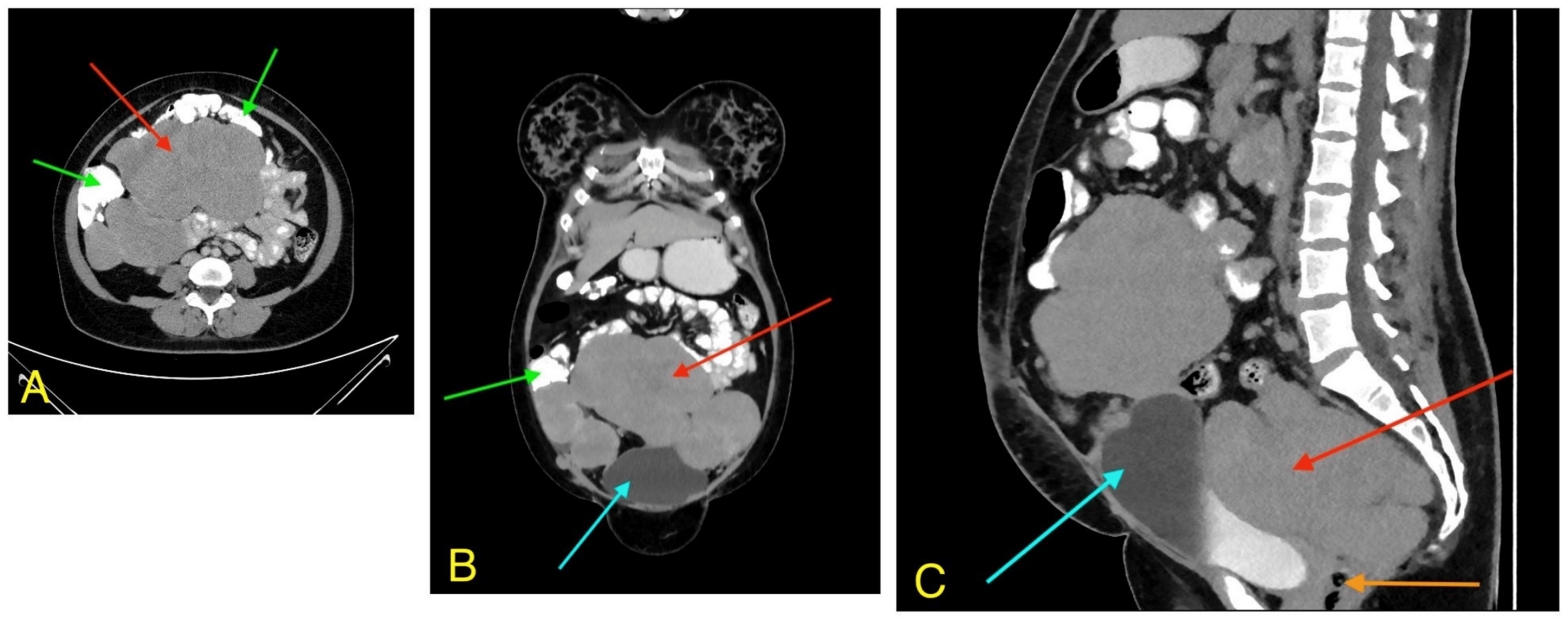
CT scan images of the abdomen – the axial (A), coronal (B), and sagittal (C) sections CT, Computerized tomography. (A) - The axial section shows the small bowel (green arrows) splayed by the large tumor (red arrow) occupying the whole of the abdomen. (B) - The coronal section shows a large central tumor (red arrow) pushing the small bowel (green arrow) up and out and abutting the urinary bladder (blue arrow). (C) - The sagittal section shows the pelvic component of the tumor (red arrow) pushing the urinary bladder (blue arrow) in the front, and compressing the rectosigmoid junction (orange arrow).

Computerized tomography (CT) scan of thorax, abdomen and pelvis was done. Multiple lesions of homogeneous density with lobulated margins and mild enhancement, post contrast, were noted in the mid and lower abdomen as well as in the pelvis. The lesions were located in multiple areas: within the peritoneal mesentery, behind the right ascending colon, in front of the right psoas muscle, and behind the urinary bladder. They also compressed and displaced the sigmoid colon and rectum to the left side, extended along the surface of the bladder dome, and were found within the omentum in the lower abdomen (Figures 1A-1C).

The largest lesion measured 17 cm x 10 cm and was in the mid-abdomen within the mesentery. The bowel loops were displaced by the multiple masses. The right lower ureter was compressed by the pelvic mass with proximal grade one hydroureter and hydronephrosis. A small nodule of 1.2 cm was seen involving the anterior pelvic wall in the right iliac fossa. No significant calcification or necrosis was noted in the above-mentioned masses except for a small amount of necrosis in the mass along the dome of urinary bladder. The ovaries could not be seen. There was a 2.8 cm x 2.3 cm sized, well-defined, smooth-walled cystic lesion seen in the right iliac fossa. No other abnormality was noted in the thorax and the liver. 

The patient underwent upper gastrointestinal endoscopy and colonoscopy, which did not show any mucosal lesion. The option of image-guided biopsy was discussed, but due to the high risk of bowel injury, it was not considered. The case was discussed in the multidisciplinary board meeting. The possibility of a malignancy, including malignancy of ovarian origin, gastrointestinal stromal tumor (GIST), sarcoma, and mesothelioma was considered as differential diagnoses. A remote possibility of peritoneal leiomyomatosis was also discussed, due to the past history of laparoscopic hysterectomy for myoma. 

Surgical findings

**Figure 2:**
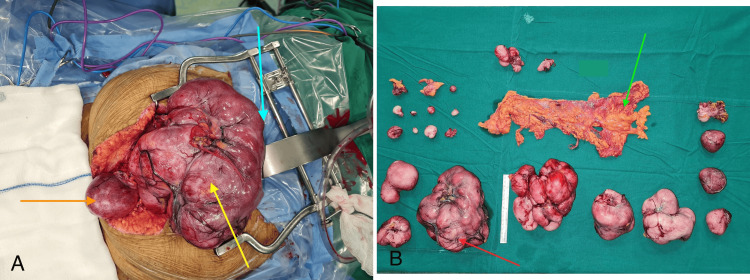
Surgical findings – Mid-line laparotomy (A) and surgical specimens (B) Blue arrow – Cranial extent Orange arrow – Caudal extent Yellow arrow – Tumor Red arrow – Largest lesion Green arrow – Omentum

The patient was taken up for surgery under general and epidural anesthesia. Preoperative ureteric catheters were placed as the pre-operative imaging showed hydroureteronephrosis and the possibility of ureteric encasement. During the surgery, there was extensive nodular disease involving the peritoneum in the abdomen, pelvis, omentum, and the mesentery of the small bowel (Figure 2A).

A total of 23 nodules of varying sizes were noted during the surgery. The largest lesion was about 19 cm in diameter. A minimal ascites of about 100 ml was noted, which was sent for cytology. All the lesions were excised along with the omentum (Figure 2B).

The ovaries were removed as they were entangled by the disease in the pelvis. The urinary bladder was carefully dissected from the nodules. Significant adhesions were noted in the pelvis due to the previous surgery. Both ureters were identified and dissected. There were no significant nodes in pelvis or retroperitoneum and nodal sampling was done from these regions. The disease was completely resected without any gross residue at the end of the surgery. Ureteric catheters assisted in identifying the ureter during the surgery and avoiding iatrogenic trauma to the ureter. A frozen section was not utilized as it was unlikely to change the plan of surgery. Blood transfusion was not needed during the perioperative period.

The patient was stable throughout the procedure and the ureteric catheters were removed at the end of it. She recovered well, was started on oral diet on the second day after the operation, and was discharged on the fifth day. The surgical wound healed well.

Histopathology

**Figure 3:**
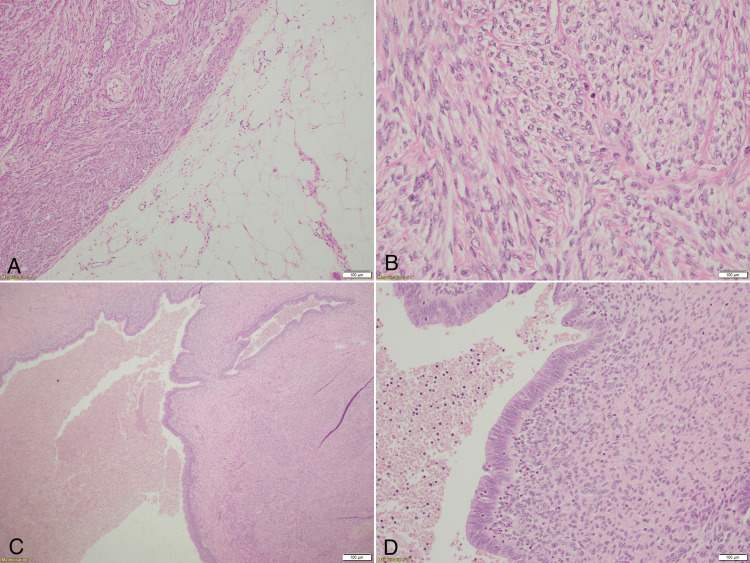
Histologic sections stained with hematoxylin and eosin (H&E) (A) – Peritoneal leiomyomatous nodule surrounded by mature adipocytes (4x magnification). (B) - Leiomyomatous nodule with proliferating benign spindle-shaped smooth muscle cells (40x magnification). (C) - Extrauterine adenomyoma highlighting two dilated benign endometrial glands surrounded by smooth muscle cells (20x magnification). (D) - Extrauterine adenomyoma highlighting two dilated benign endometrial glands lined by columnar epithelium, and a few spindle-shaped endometrial stromal cells noted around the gland (40x magnification).

Histopathology showed peritoneal leiomyomatosis (Figures 3A, 3B) and extrauterine adenomyoma (Figures 3C, 3D). The omentum showed a single reactive lymph node.

The gross picture showed grayish-white masses with intact surface capsules. The cut section was grayish white, solid, and firm in texture.

Microscopy showed fusiform structure with smooth muscle cells. The spindle cells were uniform in size. There was no atypia or invasive growth pattern. The mitotic count was low at less than five per 10 high power field (HPF). The ovaries showed hemorrhagic corpus luteal cysts. Ten nodes from the pelvis and the retroperitoneum showed reactive changes. The ascitic fluid cytology showed no atypical cells. Further immunohistochemistry was not done as the above picture (Figure 3) confirmed disseminated peritoneal leiomyomatosis (DPL). The option of hormonal management was discussed, but the patient preferred to be on close follow up.

Upon follow-up, it was determined that she had been free of disease recurrence for three years and the imaging done in October 2024 did not show any recurrence.

## Discussion

DPL is an uncommon condition that can occur in 0.35% of patients after laparoscopic morcellation [2]. It is also called leiomyomatosis peritonealis disseminate. The incidence ranges between 0.12%-0.95% [1,3]. DPL presents as multiple smooth muscle tumors of variable sizes along the peritoneal surface. It was first documented in 1952 by Wilson and Paele [4]. The nomenclature was first documented by Taubert et al. in 1964 [4,5]. It is usually seen in females in the menstrual age group. Typically, familial clustering of cases is not seen except for one study where six persons in a family had DPL, and three of them were male [6]. This was an exception. Otherwise, there has been no pattern identified in families.

Pathogenesis

DPL is documented in young females in the premenstrual age group with an average time interval of 39-132 months after surgical procedures like laparoscopic hysterectomy or myomectomy. There are various postulates in the evolution of DPL. Iatrogenic theory suggests uncontrolled morcellation during laparoscopic procedures result in the seeding of uterine leiomyomas into the peritoneal cavity and surface of viscera, due to spillage [1,2,7,8]. The term "parasitic leiomyoma" is also used to describe the underlying cause [9]. Parasitic leiomyoma was also postulated to arise from subserosal fibroids. These fibroids adhere to adjacent structures (broad ligament or omentum) to derive vascular supply, in turn, losing their attachment to the uterus. They were hence termed as parasitic leiomyomas [8,10]. Apart from spillage, other pathogenic factors proposed are genetic predisposition, hormonal factors, and peritoneal mesenchymal stem cell metaplasia. Multiple factors come into a complex interplay resulting in DPL. About 200 cases of DPL have been reported in the literature [5]. The influence of hormones in the evolution of DPL has been documented in many studies. DPL has been associated with pregnancy, long-term use of oral contraceptives or hormone replacement therapy, and estrogen-secreting tumors of the ovary [7,10]. Cytogenetic studies showing monoclonal cells of origin, possibly suggest metastatic scattering of unicentric disease from UL [7]. The multicentric presentation of these benign tumors of Mullerian origin is differentiated from sarcoma only by histopathology [11]. Due to the rarity of these cases, the etiology remains elusive. Apart from the smooth muscle element, rarely, extra-uterine adenomyomas composed of smooth muscle cells, endometrial glands, and endometrial stroma have been documented in histopathology [1,12].

Imaging and evaluation

In our case, the patient had a laparoscopic hysterectomy following which she developed the present problem in three years. The symptoms were also quite vague. In most cases, DPL is asymptomatic. It can be noticed incidentally during evaluation for unrelated issues. The symptoms can vary and may include a mass in the abdomen due to the large size of the lesion, abdominal distention, discomfort from compression of adjacent organs, and vague abdominal pain. There are no serum markers specific for DPL. Due to rarity of the disease, diagnosis is usually made after the final histopathology. The usual differential diagnoses considered include GIST, mesothelioma, endometriosis, metastatic ovarian malignancy, and primary peritoneal carcinomatosis. The presence of necrosis, degeneration in the nodules, and heterogenous enhancement in imaging might suggest a malignant tumor [13,14]. In our case, the lesions in the CT scan were homogenous in appearance. Some of them were massive and occupied a large area. There was no necrosis or degeneration. Magnetic resonance imaging (MRI) adds value to the evaluation if there is a doubt of sarcomatous degeneration [14]. The characteristic T1- and T2-hypointense signals, resembling those of smooth muscle in uterine leiomyomas, along with variable post-contrast enhancement, suggest a benign etiology [10].

Surgical treatment

As there is no documentation available on spontaneous resolution, in a medically-fit patient, the only way to achieve a cure for DPL is by complete surgical resection [1]. On a few occasions, due to the extensive nature of recurrence, multiorgan complex resections have been done to clear the disease. This might lead to extensive adhesions and possible short gut syndrome in the due course, if long segments of bowel are resected. A multidisciplinary team is required during surgery. For patients with limited disease and those wanting to preserve their fertility, focal resection of the lesions with conservation of the uterus and the ovary can be done. For patients with extensive disease and those not requiring fertility preservation, resection of all the masses with the uterus, bilateral adnexa, and omentum should be considered as the optimal surgical procedure. In our case, the patient had extensive disease and all the disease was removed along with the ovaries and omentum. There was no need for multiorgan resection in our case. Care must be taken to strike a balance between the complete removal of the disease and minimizing organ resection, ensuring that the ultimate goal of total surgical resection is not compromised.

Adjuvant therapy

Histopathological examination of the specimen remains the cornerstone in confirming the diagnosis of DPL and ruling out the rare possibility of malignant sarcomatous degeneration [15]. Under microscopy, DPL classically does not have necrosis, mitotic activity and cellular atypia with a low marker of proliferation Kiel 67 (Ki 67) index. The role of the frozen section has been limited by the small number of cases. One case report showed benign changes in the frozen section which limited the surgery to a minimal level, to remove all the gross disease and avoid multiorgan resection [16]. As DPL is not a malignancy, aggressive treatment like chemotherapy need not be considered. In selected cases, a gonadotropin releasing hormone (GnRH) analog or an aromatase inhibitor [9] can be considered to suppress the hormonal stimulation.

Preventive method

It is imperative to ensure that the peritoneal dissemination does not happen at the time of morcellation of the specimen during the primary procedure of laparoscopic hysterectomy for uterine fibroids. Technical precautions should be observed to prevent spillage, including the use of an endo-bag technique for confined laparoscopic morcellation (LM), which may help reduce the risk of the debilitating conditions such as DPL [14].

Malignant transformation

The chance of malignant transformation of DPL is rare. The risk is about 2-5% as reported in the literature [15]. There are ongoing studies aimed at better understanding the potential for malignant transformation. The role of epithelial-to-mesenchymal transition (EMT) activating transcription factors in driving sarcomatous changes is receiving increasing attention in research. If proven, it could serve as a potential marker for malignant transformation in DPL.

## Conclusions

DPL is a rare entity. It poses a diagnostic challenge due to the extensive nature of the disease presentation. A previous history of laparoscopic myomectomy or hysterectomy for UL should raise the index of suspicion. Care should be taken to avoid spillage of tissue during morcellation and delivery of the specimen during laparoscopic myomectomy or hysterectomy. Intervention should be planned carefully for DPL to resect the disease completely with minimal damage to other viscera. While in most cases the disease can be resected by meticulous dissection, multiorgan resection should be reserved for extreme cases. This rare entity should be kept in mind while evaluating a patient with multiple abdominal masses mimicking a metastatic neoplasm.
